# Supplementary figures and images for: Mucosal immunoglobulins protect the olfactory organ of teleost fish against parasitic infection
Source: PLoS Pathog. 2018 Nov 5;14(11):e1007251. doi: 10.1371/journal.ppat.1007251 (PMC6237424; doi:10.1371/journal.ppat.1007251)

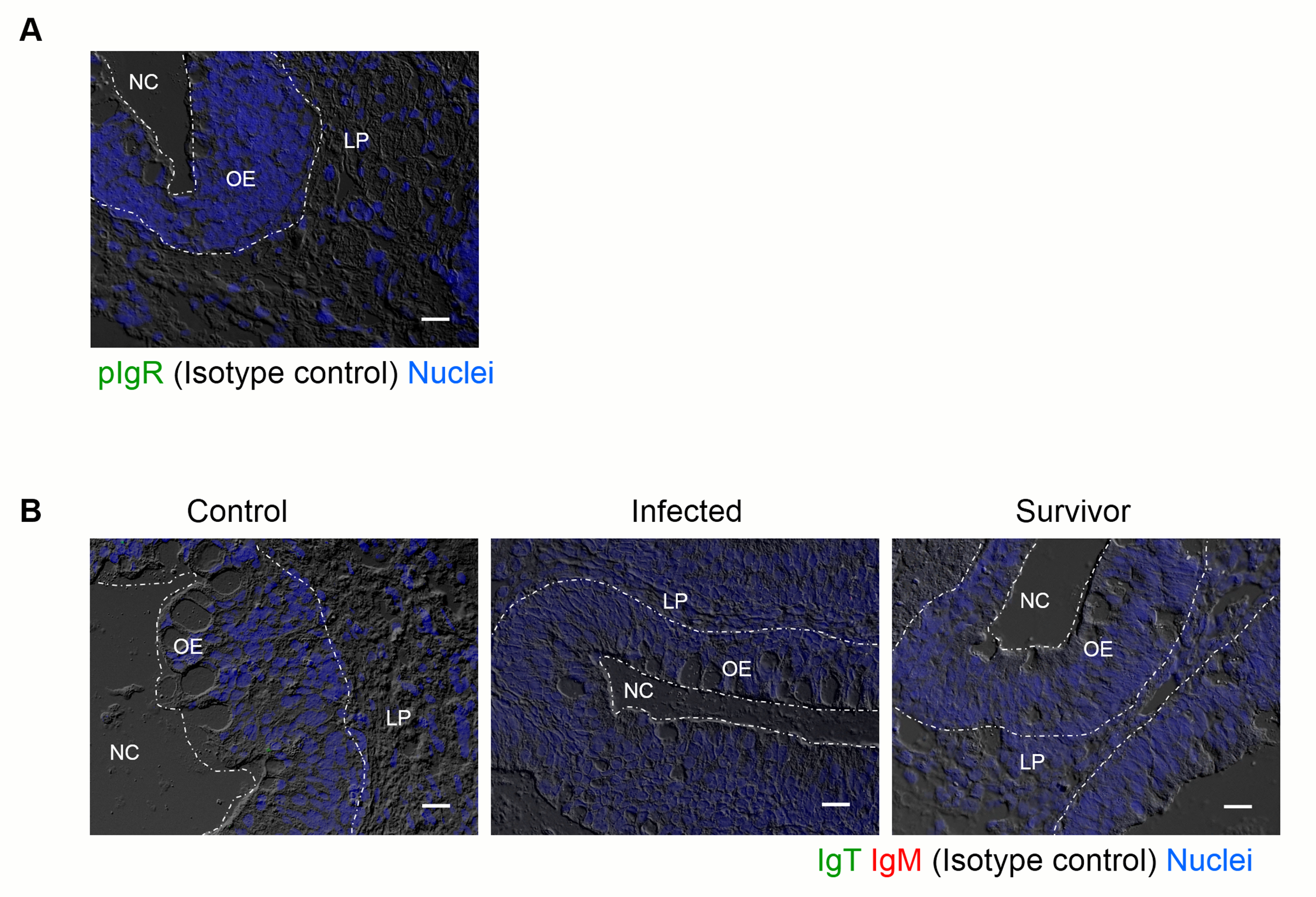

Supplement: S1 Fig — Differential interference contrast images of olfactory organ paraffin sections from 28 days Ich-infected fish (A middle and B), survivor fish (A right), and control fish (A left), with merged staining of isotype control antibodies for anti-trout IgT (green) or anti-trout IgM mAbs (red) (A); or for anti-trout pIgR pAb (green, B). Nuclei were stained with DAPI (blue, A and B). NC, nasal cavity; OE, olfactory epithelium; LP, lamina propria. Scale bar, 20 μm. Data are representative of at least three different independent experiments. (TIF) [file ppat.1007251.s001.tif]

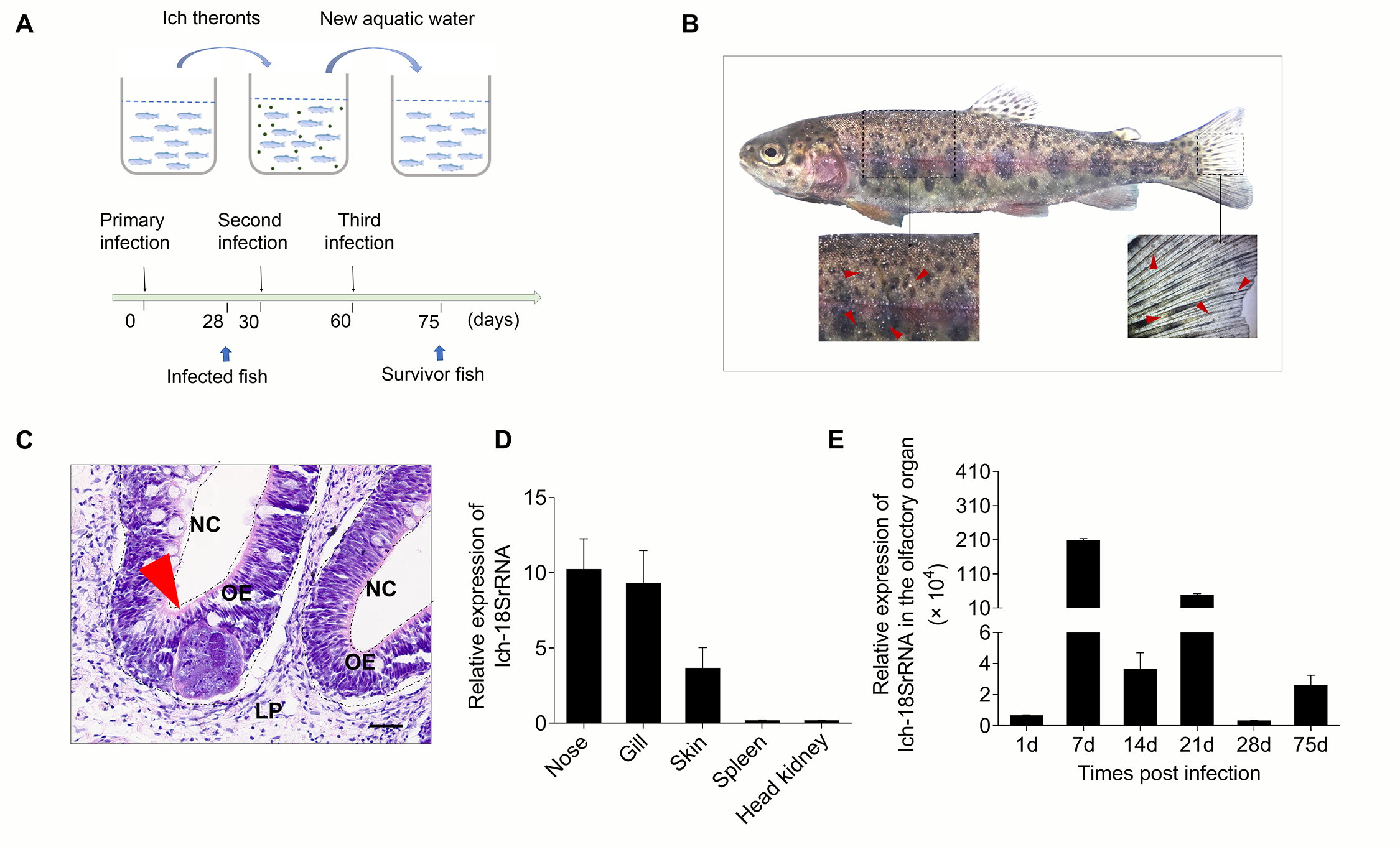

Supplement: S2 Fig — (A) Infected method with Ich parasite by bath has been used in this study. (B) The phenotype of rainbow trout was observed at 7 days post infection with Ich (n = 12). The red arrows represent the obvious while dot in skin (lower, left) and fin (lower, right). (C) Histological studies of olfactory organ from 7 days Ich-infected trout by staining with haematoxylin / eosin (H & E). Results are representative of one experiment n = 6. Scale bar: 50 μm. (D) The relative expression of Ich-18SrRNA gene in olfactory organ, gills, skin, spleen and head kidney from 7 days Ich-infected trout. (E) The relative expression of Ich-18SrRNA gene in olfactory organ at 1, 7, 14, 21, 28 and 75 days post infection. Data in d and e are representative of at least three independent experiments (mean and s.e.m.). Statistical analysis was performed by unpaired Student’s t-test. *P < 0.05, **P < 0.01 and ***P < 0.001. (TIF) [file ppat.1007251.s002.tif]

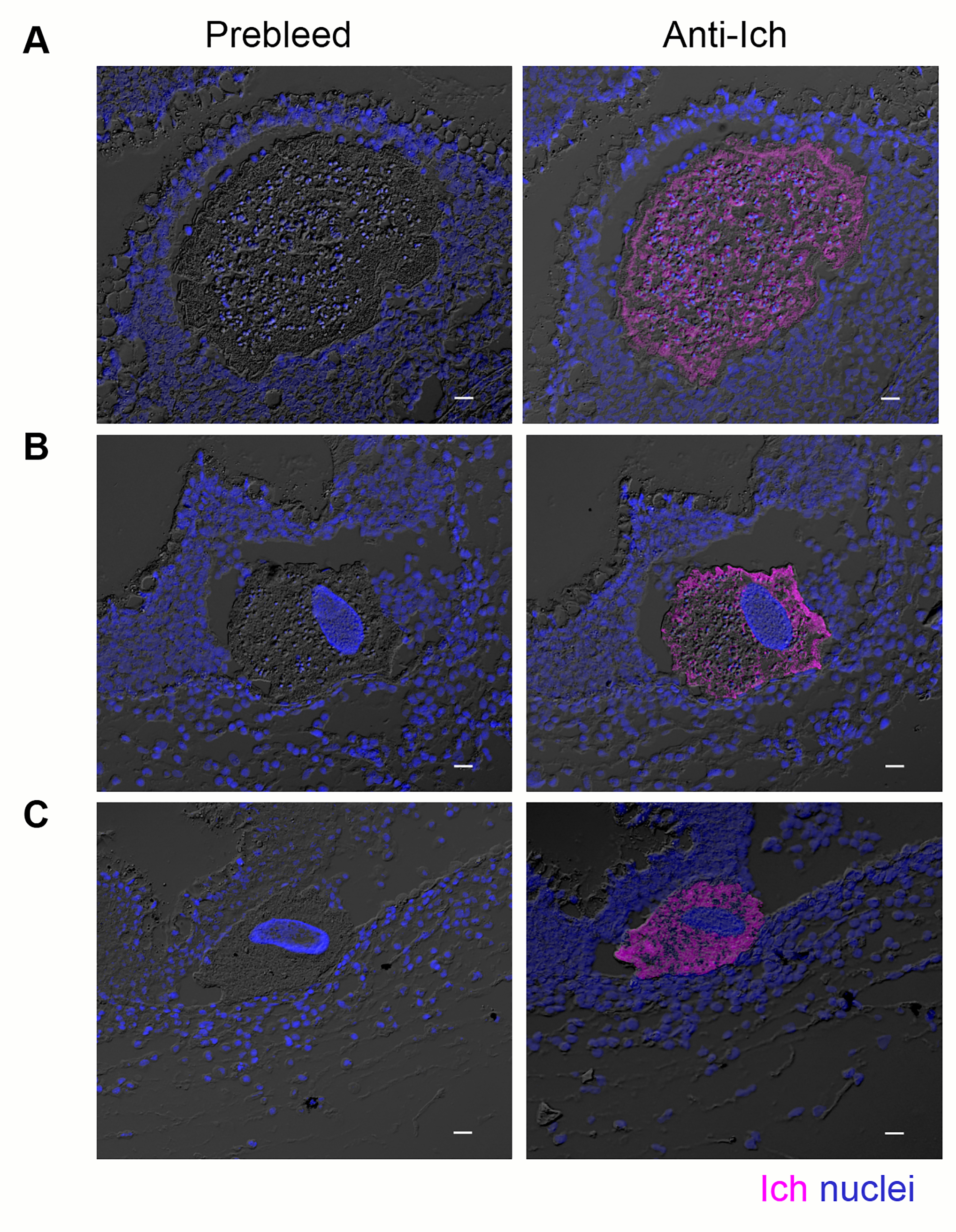

Supplement: S3 Fig — Three different microscope images of consecutive slides of prebleed (A-C left) and anti-Ich (A-C right) antibodies staining of Ich parasite in olfactory organ paraffin sections from 28 days Ich-infected fish (n = 4). Nuclei were stained with DAPI (blue) and Ich with anti-Ich pAb (magenta). Scale bars, 20 μm. Data are representative of three independent experiments. (TIF) [file ppat.1007251.s003.tif]

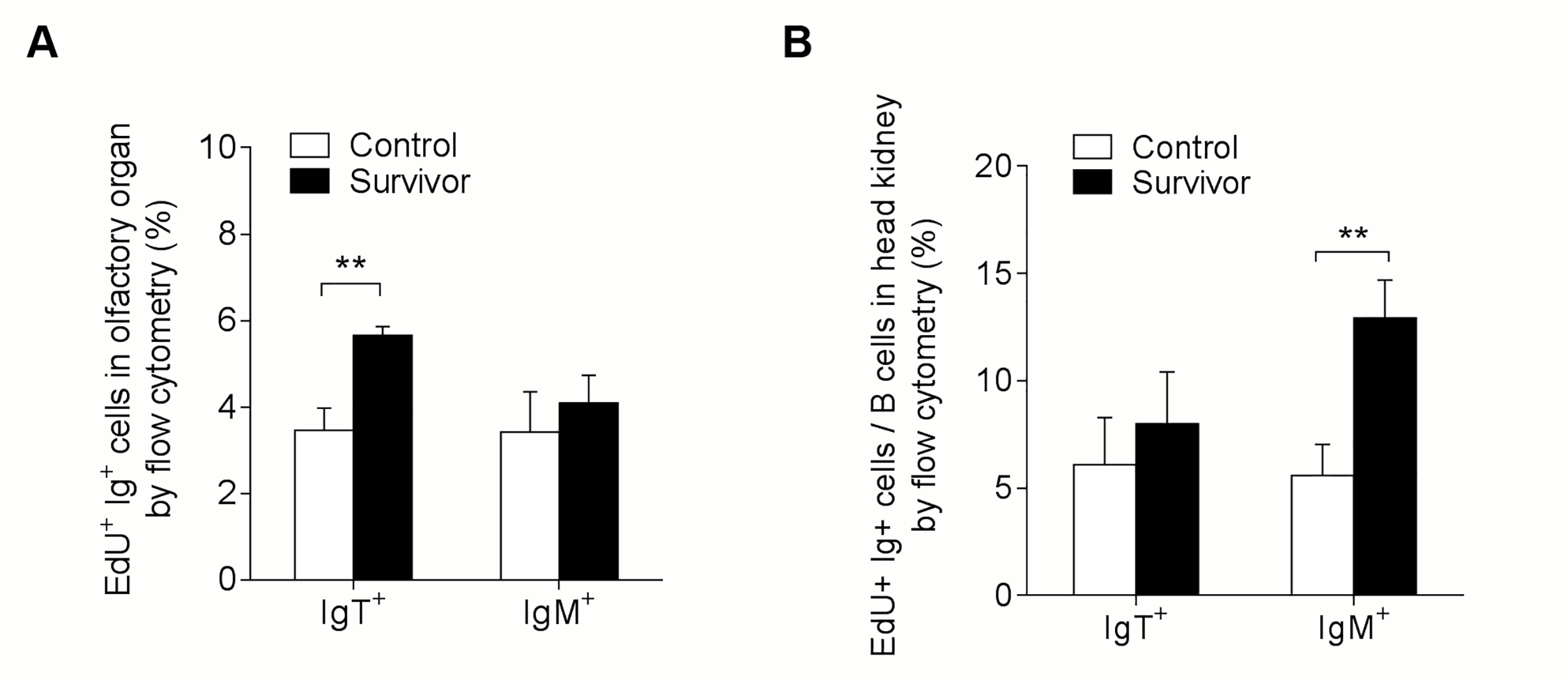

Supplement: S4 Fig — (A and B) Percentage of EdU+ cells from total olfactory organ and head kidney IgT+ and IgM+ B cell populations in control and survivor fish by flow cytometry analysis (n = 9). Data are representative of at least three different independent experiments (mean and s.e.m). Statistical analysis was performed by unpaired Student’s t-test. *P < 0.05, **P < 0.01 and ***P < 0.001. (TIF) [file ppat.1007251.s004.tif]
