# Supplementary material for: Mucosal immunoglobulins protect the olfactory organ of teleost fish against parasitic infection
Source: PLoS Pathog. 2018 Nov 5;14(11):e1007251. doi: 10.1371/journal.ppat.1007251 (PMC6237424; doi:10.1371/journal.ppat.1007251)
Supplement: S1 Table — (DOCX) [file ppat.1007251.s005.docx]

**Sup Tab 1. List of primers for real-time quantitative PCR amplifications.**

| Primer  name | Primer sequence (5'- 3') | Accession  number |
| --- | --- | --- |
| IL6 | F: ATTTCATCGTTCTCACAGC | [CCV01624.1](https://www.ncbi.nlm.nih.gov/protein/469402135?report=genbank&log$=prottop&blast_rank=2&RID=F0HEZSDB016) |
|  | R: ACTACCTCAGCAACCTTCA |  |
| C3-3 | F: TGCATGGGATCGCTAAAAGTG | U61753 |
|  | R: CCAATGACAGACAGGGTGACTTC |  |
| C3-1 | F: GAGATGGCCTCCAAGAAGATAGAA | L24433 |
|  | R: ACCGCATGTACGCATCATCA |  |
| IL11 | F: CAGAGCGTCAAGGAAACAC | NM_001124382.1 |
|  | R: GCTCCTGGGAAGACTGTAA |  |
| IL22 | F: CATCCTGGACTTCTACCTG | NM_001164064.1 |
|  | R: CCATCTCGGACAACTTCTT |  |
| C7-1 | F: GTCTATGTGGAGTTACAGGCTA | NM_001124618.1 |
|  | R: ACTTCTGCGATACGGATT |  |
| C1QL2 | F: GTCTACTCAAACATCGGC | XM_021624859.1 |
|  | R: CATTCTTGGTCAAACACAC |  |
| CLEC4E | F: GCAGCCACCTTACCATC | XM_021562202.1 |
|  | R: CACCCATCTCCAATCCC |  |
| IL10 | F: CACCGCCTTCTCCACCATC | NM_001245099.1 |
|  | R: CCATAGCGTGACACCCCAC |  |
| CXCL9 | F: GTTTCCCTCGCCACTTCAA | NP_001268281.1 |
|  | R: GCCACCCACTTGCTCTTTG |  |
| CASP8 | F: TGGTGGCAAAGGAGTTAT | XM_021588999.1 |
|  | R: CAGGAAATCGGCATCAGC |  |
| pIgR | F: AGAAGCGTTGGTGTCGTA | FJ940682.1 |
|  | R: AAGCCTTGGTCAGGTCAT |  |
| C1QBP | F: CCGCAGTCCGAATTTCTA | XM_021617398.1 |
|  | R: GCTTTGTCTCCTTCCGTAT |  |
| CSF3R | F: GGGAGGAGATTCCACTATGC | NM_001124402.1 |
|  | R: TGACAGCCCAACACCAGA |  |
| CATHL1 | F: CTGGAGGCAAGCAACAAC | AY382478.1 |
|  | R: CCCCCAAGACGAGAGACA |  |
| IgT | F: CAGACAACAGCACCTCACCTA | AY870264 |
|  | R: GAGTCAATAAGAAGACACAACGA |  |
| IgM | F: AAGAAAGCCTACAAGAGGGAGA | OMU04616 |
|  | R: CGTCAACAAGCCAAGCCACTA |  |
| MLP | F: GCTCTACTATCCAGCCAAC | XM_021578564.1 |
|  | R: GCATCCACAGTCACGAAC |  |
| MHCIIA | F: GGGTGAGTTTGTTGGATAC | DQ246664.1 |
|  | R: AGCGTTAGGCTTACATAGA |  |
| CD22 | F: TGAAGATGACAGTGGCAGAT | XP_014056970.1 |
|  | R: GGAGGGTTACAGGTGGAG |  |
| M-CSFR | F: CCCGCCTGTCACCCAATCT | AB091826 |
|  | R: CGTCCCACCAATGCTTCT |  |
| CCL25 | F: CGTGCCTGCTTGTAATG | XM_021603672.1 |
|  | R: GGGATGTGGGAAATGTC |  |
| C1S | F: AACAAGCCAATGGTTTTCAC | KKF27649.1 |
|  | R: GATTCCTTTCCCAGTTCACA |  |
| CSF1R | F: GTGAAGGAGGGCAGTGAT | NM_001124738.1 |
|  | R: GATGGTGGCAAACGCAAG |  |
| IgD | F: CAGGAGGAAAGTTCGGCATCA | JN173049.1 |
|  | R: CCTCAAGGAGCTCTGGTTTGGA |  |
| EF1α | F: CAACGATATCCGTCGTGGCA | NM_001124339.1 |
|  | R: ACAGCGAAACGACCAAGAGG |  |
